# Surface Decoration of ZnWO_4_ Nanorods with Cu_2_O Nanoparticles to Build Heterostructure with Enhanced Photocatalysis

**DOI:** 10.3390/nano8010033

**Published:** 2018-01-09

**Authors:** Lingyu Tian, Yulan Rui, Kelei Sun, Wenquan Cui, Weijia An

**Affiliations:** College of Chemical Engineering, North China University of Science and Technology, Tangshan 063210, China; Tian165162@126.com (L.T.); tsruiyulan@163.com (Y.R.); sunkelei1986@163.com (K.S.); wkcui@163.com (W.C.)

**Keywords:** nanoparticles, ZnWO4, degradation, photocatalysis

## Abstract

The surface of ZnWO_4_ nanorods was decorated with Cu_2_O nanoparticles (Cu_2_O/ZnWO_4_) prepared through a precipitation method. The Cu_2_O nanoparticles were tightly deposited on the ZnWO_4_ surface and had average diameters of 20 nm. The nanoparticles not only promoted the absorption and utilization of visible light but also facilitated the separation of photogenerated charge carriers. This brought an improvement of the photocatalytic activity. The 5 wt % Cu_2_O/ZnWO_4_ photocatalyst displayed the highest degrade efficiency for methylene blue (MB) degradation under visible light, which was 7.8 and 2 times higher than pure ZnWO_4_ and Cu_2_O, respectively. Meanwhile, the Cu_2_O/ZnWO_4_ composite photocatalyst was able to go through phenol degradation under visible light. The results of photoluminescence (PL), photocurrent, and electrochemical impedance spectra (EIS) measurements were consistent and prove the rapid separation of charge, which originated from the match level structure and the close contact with the interface. The radical and hole trapping experiments were carried out to detect the main active substances in the photodegradation process. The holes and ·O_2_^−^ radicals were predicted to dominate the photocatalytic process. Based on the characterization analysis and experiment results, a possible photocatalytic mechanism for enhancing photocatalytic activity was proposed.

## 1. Introduction

Photocatalytic technology can directly use solar energy to degrade organic pollutants using super oxidation capacity, mild reaction conditions, and no secondary pollution. The development of an efficient semiconductor photocatalyst plays an important role in the practical application of photocatalytic technology [1]. Recently, ZnWO_4_ received widespread attention due to its excellent performance in degrading organic pollutants under ultraviolet light [2,3]. Meanwhile, the ZnWO_4_ photocatalyst also has the advantages of stable chemical properties and is easy to develop. However, the pure ZnWO_4_ photocatalyst suffers from a higher photogenerated charge recombination rate and a low utilization rate of sunlight, which severely limits its practical application ability [4,5]. Therefore, the key problem for improving the photocatalytic performance of ZnWO_4_ is to boost the rapid separation of photo-generated electron-hole pairs on the basis of efficient utilization of solar energy.

Noble metal decorated ZnWO_4_ can promote the rapid separation of photogenerated charge through the schottky barrier since the Fermi level of noble metal is more positive than ZnWO_4_ [5,6]. Additionally, ion doping, such as seen in fluorine and chlorine [7,8,9,10], could greatly enhance photocatalytic performance since the doped atoms can act as electron traps and promote photogenerated charge separation. In addition, construction of heterojunction photocatalyst by combining ZnWO_4_ with other semiconductors, such as Bi_2_WO_6_/ZnWO_4_ [11], ZnWO_4_/BiOI [12], and BiOBr/ZnWO_4_ [13], provides a feasible route to facilitate the effective transmission of the charge through interfacial interfaces. Additionally, by combining ZnWO_4_ with material that has a π–π conjugated structure like C_3_N_4_ [14,15] and graphene [16] could effectively inhibit the combination of photo-generated carriers due to the special conductivity of the conjugate material. Recently, Niu [17] and Li [18] prepared Ag/AgCl and Ag@AgBr nanoparticles decorate ZnWO_4_ nanorods, respectively, to obtain more visible light utilization efficiency for the surface plasmon resonance of Ag nanoparticles. In addition, surface modification of ZnWO_4_ with narrow band-gap and matching energy level semiconductors not only improved the visible light response but promoted the separation of the photogenerated charges. 

As commonly known, Cu_2_O can absorb the most visible light for the band gap at about 2.2 eV [19] when using Cu_2_O nanoparticles modified for the wide bandgap semiconductor. Modifications such as TiO_2_ [20], BiVO_4_ [21], and BiOBr [22] can greatly enhance the absorption of visible light on the composite. Moreover, based on the morphology, the grain size of the catalyst has an important influence on the activity. Various morphologies of Cu_2_O can be prepared through multiple methods [23] and exhibit excellent photocatalytic performance. Some studies have pointed out that Cu_2_O nanoparticles were recently modified on the TiO_2_ nanosheets or multi-walled carbon nanotubes not to enhance the light absorption but to improve the separation of the photogenerated carriers. This generates a higher photocatalytic performance [24,25]. Considering the criteria, this action loaded the Cu_2_O nanoparticles onto the surface of the ZnWO_4_ nanorods to further improve the utilization rate of visible light and then to stimulate production of more electron–holes. Meanwhile, the match between the level structure and the intimately contacted interface of ZnWO_4_ and Cu_2_O are beneficial for accelerating the separation of the photogenerated charge carriers, which improves the photocatalytic activity. In fact, there are no previous studies about using Cu_2_O nanoparticles to decorate ZnWO_4_ nanorods.

Herein, we modified Cu_2_O nanoparticles onto the surface of ZnWO_4_ nanorods. The Cu_2_O nanoparticles were tightly deposited on the ZnWO_4_ surface with average diameters of 20 nm. Our results proved that the Cu_2_O nanoparticles greatly promoted the absorption and utilization of visible light. Meanwhile, the introduction of Cu_2_O nanoparticles provides a new channel for charge transfer. This greatly inhibited photo-generated carrier recombination and accelerated the migration of interfacial charge carriers. The effects of different Cu_2_O nanoparticles content on the visible light absorption and charge separation of the composites were investigated. The 5 wt % Cu_2_O/ZnWO_4_ photocatalyst displays the highest degrade efficiency for methylene blue (MB) degradation, which was 7.8 and 2 times more than seen in pure ZnWO_4_ and Cu_2_O. Based on the characterization analysis and experiment results, a possible photocatalytic mechanism on enhancement of photocatalytic activity was proposed.

## 2. Experimental

### 2.1. Synthesis of Photocatalysts

The catalyst is prepared with deionized water. The activity test process uses ultrapure water. All of the reagents are analytically graded. Initially, the synthesis of ZnWO_4_ was carried out using the hydrothermal method based on previous work [26]. Typically, Na_2_WO_4_·2H_2_O (3 mmol) and Zn(NO_3_)_2_·6H_2_O (3 mmol) were dispersed in 25 mL distilled water with magnetic stirring for 15 min. Then, the two above aqueous solutions were mixed together through magnetic stirring at room temperature for 30 min. Afterward, the mixture was transferred into a Teflon-lined steel autoclave and the autoclaves were heated in a convection oven at 180 °C for 12 h. The resulting precipitates were collected and washed with deionized water and absolute ethanol. The samples were then dried at 80 °C for 10 h.

Then, Cu_2_O/ZnWO_4_ composite was prepared using a simple reductive solution chemistry route based on a previous report [22]. First, measured amounts of cetyl trimethyl ammonium bromide (CTAB) and ethylene diamine tetra acetic acid (EDTA) were dissolved in 50 mL deionized water and stirred until a stable suspension was obtained. Then, 0.5 g ZnWO_4_ was added into the uniform suspension and stirred for 30 min, followed by the addition 0.06 g Cu(Ac)_2_. Thirty minutes later, 25 mL NaOH solution (0.45 mol·L^−1^) was added dropwise to the mixed solution. The color of the solution became blue, demonstrating the formation of Cu(OH)_2_. After 30 min, the solution color slowly turned to orange by adding 25 mL of ascorbic acid (AA) (0.3 mol·L^−1^) solution dropwise. Finally, composites were washed with ethanol and water and dried at 60 °C for 6 h to obtain Cu_2_O/ZnWO_4_. 

### 2.2. Characterization

The crystal structure and phase analysis of the Cu2O/ZnWO4 composite was analyzed through X-ray diffraction (XRD) using a Rigaku D/MAX2500 PC diffractometer (D/MAX2500 PC, Rigaku Corporation, Tokyo, Japan) with Cu Kα radiation, with an operating voltage of 40 kV and an operating current of 100 mA. The morphology and particle size of the catalyst were observed through transmission electron microscopy (TEM, JEM-2010, JEOL Ltd., Akishima, Japan). The UV-visible light (UV-Vis) was used to determine the light absorption properties of the catalyst (Puxi, UV1901, Beijing, China). The surface chemical state was analyzed by an X-ray photoelectron spectroscope (XPS, Shimadzu Kratos, AXIS-Ultra DLD, Tokyo, Japan) with a monochromatized Al Kα radiation source (1486.6 eV). We performed electrochemical and photoelectrochemical measurements using a three-electrode quartz (CHI-660E, Chen Hua Instruments, Shanghai, China). Photoluminescence (PL, Hitachi F-7000, Tokyo, Japan) Spectra were collected to explore the recombination of photogenerated carriers. 

### 2.3. Photocurrent and Electrochemical Impedance Spectra Measurements

The photocurrent and electrochemical impedance spectra (EIS) measurements were conducted using an electrochemical analyzer (CHI660E, Chen Hua Instruments, Shanghai, China) with a standard three-electrode configuration. A standard three-electrode cell was used in the photoelectric studies including a working electrode (as-prepared photocatalyst), a platinum wire as the counter electrode, and a standard calomel electrode (SCE) as the reference electrode. An amount of 0.1 M Na_2_SO_4_ was used as the electrolyte solution. The visible light irradiation was obtained from a 500 W Xe lamp with a 420 nm cutoff filter.

### 2.4. Photocatalytic Activity

The photocatalytic gradation of organic pollutants was carried out in a tube photoreactor. In order to eliminate the effect of temperature on the rate of photocatalytic degradation, the photochemical reaction apparatus was connected to a circulating cooling device (Bilon, Xi’an, China) to control the temperature of the reaction solution in the test tube at 25 ± 2 °C. The irradiation light source was a 400 W metal halide lamp. Adding the filter glass sheets between the metal halide lamp and the reaction tube filtered wavelengths <420 nm. The catalyst activity was analyzed primarily by degrading 10 ppm MB by 0.1 g of catalyst. After stirring for 30 min under dark, 1 mL of sample was taken every 15 min. The collected supernatant solutions were analyzed by the spectrometer at the characteristic absorption peak of 664 nm. The samples obtained during the photocatalytic test were filtered through a filter. Additionally, the degradation of phenol solution was detected by using high performance liquid chromatography (HPLC). 

## 3. Results and Discussion

**Figure 1:**
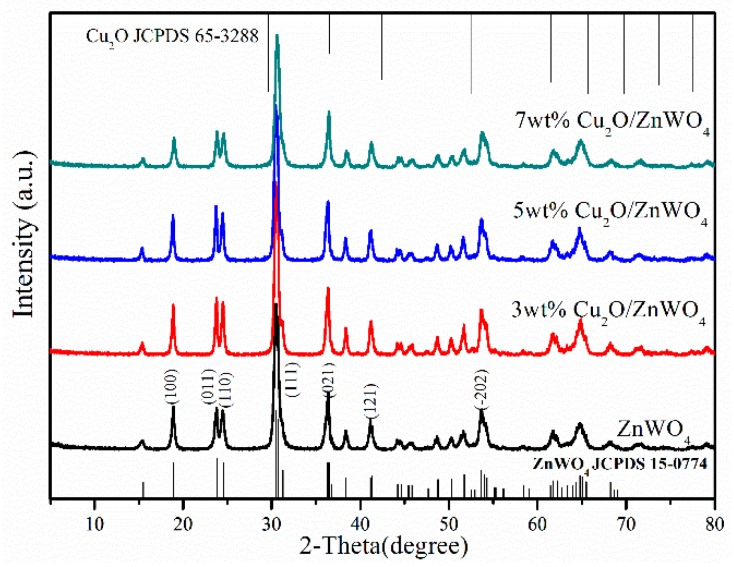
X-Ray Diffraction (XRD) patterns of ZnWO_4_ and Cu_2_O/ZnWO_4_ photocatalysts.

The crystallographic structures of ZnWO_4_ and Cu_2_O/ZnWO_4_ samples were confirmed by XRD measurements. As seen in Figure 1, the diffraction peaks were observed at 18.91°, 23.84°, 24.58°, 30.72°, 36.31°, 41.15°, and 53.63°, corresponding to the (110), (200), (210), (211), (220), (310), (222), (320), (321), (400) and (421) crystal surfaces of ZnWO_4_, respectively. This aligns with the standard card (JCPDS 15-0774) [27]. The standard card and the characteristic diffraction peaks were narrow and sharp, indicating that the composites possessed of high crystallinity. Meanwhile, the composites showed high purity since there were no traces of other phases examined. Compared with pure ZnWO_4_, the XRD patterns of the Cu_2_O/ZnWO_4_ composites did not vary in shapes or peaks and it was presumed that the addition of Cu_2_O did not change the crystal form of ZnWO_4_. Additionally, no characteristic diffraction peaks attributed to Cu_2_O were detected in the Cu_2_O/ZnWO_4_ composites (as seen the Cu_2_O JCPDS 65-3288) [22], which was caused by the small amounts and the particle size of Cu_2_O nanoparticles (max 7 wt %) as well as the high dispersion of Cu_2_O nanoparticles.

**Figure 2:**
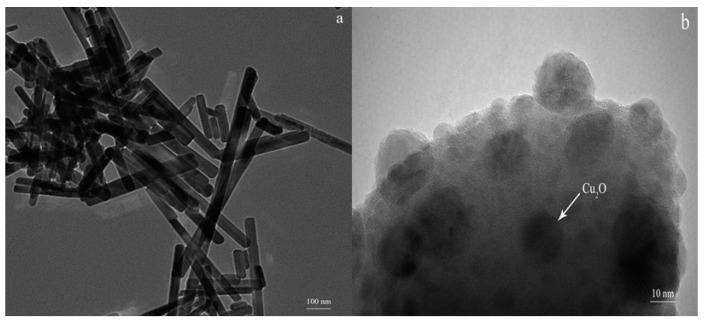
Transmission Electron Microscopy (TEM) images of (**a**) ZnWO_4_; (**b**) Cu_2_O/ZnWO_4_.

The TEM was performed on the ZnWO_4_ and 5 wt % Cu_2_O/ZnWO_4_ composite to investigate the morphology and detail structural information of the Cu_2_O/ZnWO_4_ composite catalyst. As seen in Figure 2a, the pure ZnWO_4_ nanorods have a smooth surface with a length of 200–500 nm and a width of 20–30 nm. In comparison, as seen in Figure 2b, the Cu_2_O nanoparticles, with average diameters of 20 nm, were coated on the surface of ZnWO_4_ nanorods uniformly. The Cu_2_O nanoparticles did not significantly change its morphology or the size of ZnWO_4_ photocatalysts, which was consistent with the XRD results. Meanwhile, the Cu_2_O nanoparticles were beneficial for separating the photo-generated carriers and improving the degradation performance of composites.

**Figure 3:**
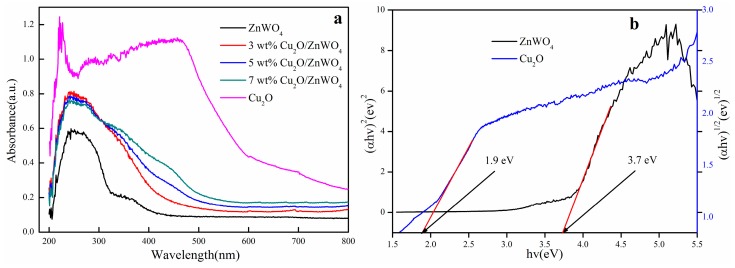
(**a**) UV-visible light (UV-vis) diffuses reflection spectra of pure ZnWO_4_, Cu_2_O and Cu_2_O/ZnWO_4_ samples; (**b**) the band gap energies of ZnWO_4_ and Cu_2_O.

**Figure 4:**
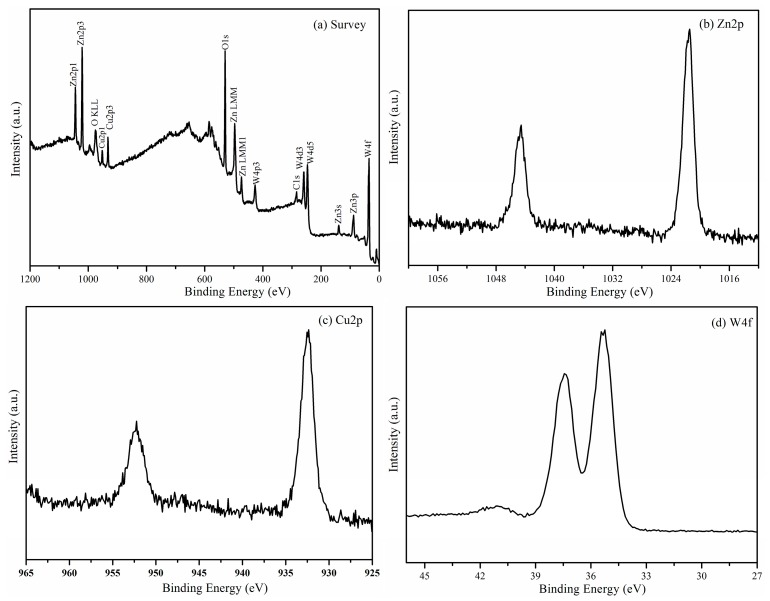
X-Ray Photoelectron Spectroscopy (XPS) spectra of Cu_2_O/ZnWO_4_ sample: (**a**) Survey of the sample; (**b**) Zn 2p; (**c**) Cu 2p and (**d**) W 4f.

Figure 3a shows the UV-visible diffuse reflectance pattern of ZnWO_4_, Cu_2_O and Cu_2_O/ZnWO_4_ samples. As seen in Figure 3a, the pure ZnWO_4_ shows its fundamental absorption edge at 320 nm due to its large band-gap energy while the Cu_2_O exhibited strong absorption in the λ < 600 nm region. When Cu_2_O nanoparticles were modified on the ZnWO_4_ surface, it brought more visible light absorption for all of the Cu_2_O/ZnWO_4_ composites, and red shifted into the visible light region because of the photosensitizing effect of the incorporated Cu_2_O nanoparticles. Additionally, the visible light absorption edge increased with rising Cu_2_O content. The forbidden band width of a semiconductor catalyst can be calculated using the Kubelka-Munk equation [28]. The result is shown in Figure 4b. According to the literature [21,29], the Cu_2_O was a direct transition semiconductor and the ZnWO_4_ was an indirect transition. As such, the band gap of pure Cu_2_O and ZnWO_4_ was calculated to be 1.9 and 3.7 eV (as seen in Figure 3b), respectively. 

So as to demonstrate the interaction between Cu_2_O and ZnWO_4_ in Cu_2_O/ZnWO_4_ composites, the valence state and the binding energy in Cu_2_O/ZnWO_4_ were determined by using XPS. The results show that the element of Cu, O, Zn, W and C all appear in the full XPS spectrum of Cu_2_O/ZnWO_4_ and the corresponding high resolution spectra, which are noted in Figure 4b–d. Zn 2p binding energy position was shown in Figure 4b. In Figure 4b, we can observe that the characteristic peaks appear at 1022.1 and 1044.5 eV, respectively, which corresponds to the peak position of Zn 2p_3/2_ and Zn 2p_1/2_ [14]. Figure 4c is the spectrum result of Cu 2p. There are two peaks shown at 931.9 and 951.9 eV, which corresponds to Cu 2p_3/2_ and Cu 2p_1/2_, respectively. The experimental results are consistent with reports in the literature [22]. The XPS spectrum of W 4f (as seen in Figure 4d) shows that two binding energy peaks appear at 35.0 eV and at 37.4 eV, respectively for W 4f_7/2_ and W 4f_5/2_, which determines the existence of W [30]. After the analysis of XRD and XPS, it can be concluded that Cu_2_O and ZnWO_4_ coexist in the Cu_2_O/ZnWO_4_ sample.

**Figure 5:**
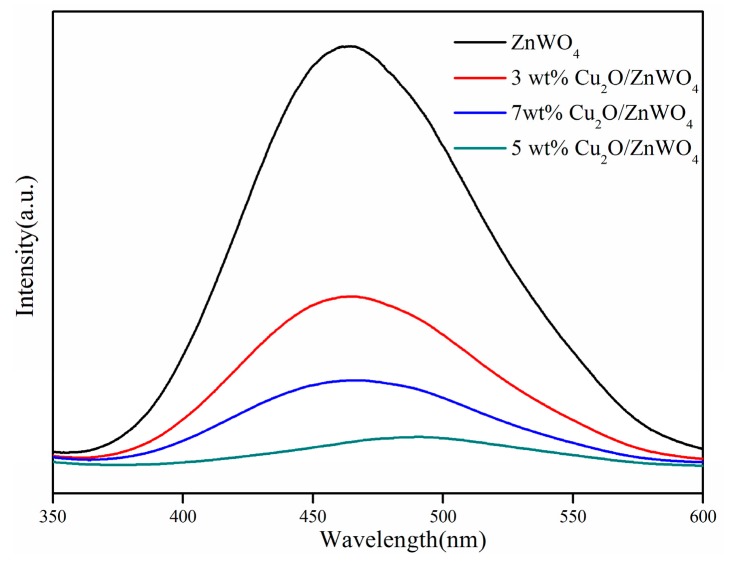
Photoluminescence (PL) spectra of ZnWO_4_ and Cu_2_O/ZnWO_4_ composites.

The photoluminescence (PL) spectra can evaluate the transfer and recombination processes of photogenerated e^−^/h^+^ pairs in semiconductors. It is widely accepted that the lower PL intensity, the lower photo-carrier recombination rate and the higher photocatalytic performance [31]. The excitation wavelength ZnWO_4_ and Cu_2_O in the Cu_2_O/ZnWO_4_ architectures was 315 nm. The comparison of PL spectra is shown in Figure 5. The main emission peak is centered at 465 nm for the ZnWO_4_ sample due to the recombination of photogenerated carriers in the ZnWO_4_. Once Cu_2_O nanoparticles were added, the photoluminescence dropped markedly and the 5 wt % Cu_2_O/ZnWO_4_ composites displayed the lowest PL intensity, which indicates that the photogenerated carriers were effectively separated in the composite semiconductors. This shows that the process was conducive to promoting the photocatalytic properties of the composite material.

**Figure 6:**
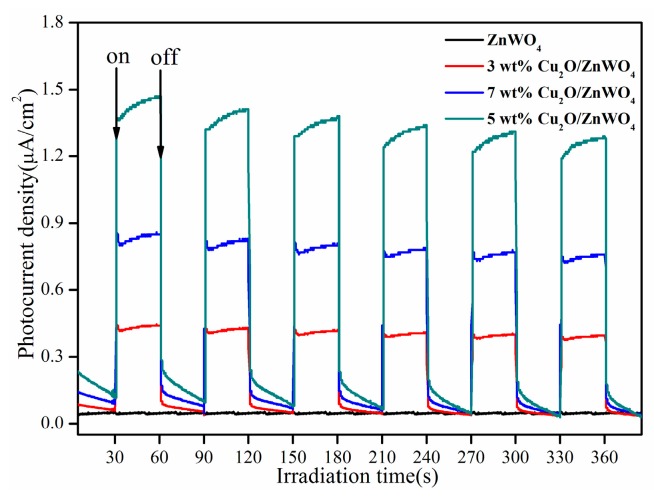
Photocurrent-time curves of bulk ZnWO_4_ and Cu_2_O/ZnWO_4_ composites under visible light (>420 nm) irradiation with 30 s light on/off cycles.

In order to better explain the photogenerated carriers, the Cu_2_O/ZnWO_4_ composites should be effectively separated. The transient photocurrent responses of the ZnWO_4_ and Cu_2_O/ZnWO_4_ composites were recorded for several on/off cycles of irradiation. It was generally considered that the higher the peak intensity, the higher the separation efficiency of the photogenerated carriers [32]. As shown in Figure 6, when visible light is irradiated, the photocurrent increases sharply. In contrast, in the dark environment, the photocurrent was immediately reduced to zero. A significant photoelectric response was observed from the on/off photoperiod. In addition, ZnWO_4_ showed almost no photocurrent response due to its lack of visible light response while the Cu_2_O/ZnWO_4_ composites displayed much higher photocurrent intensity, where the 5 wt % Cu_2_O/ZnWO_4_ composites exhibited the highest photocurrent density. This shows that the introduction of Cu_2_O nanoparticles will greatly improve the photocurrent response. The Cu_2_O/ZnWO_4_ composites exhibit faster charge separation efficiency, which originates from the ZnWO_4_ and Cu_2_O matching energy level structure that rapidly separates photogenerated hole pairs.

**Figure 7:**
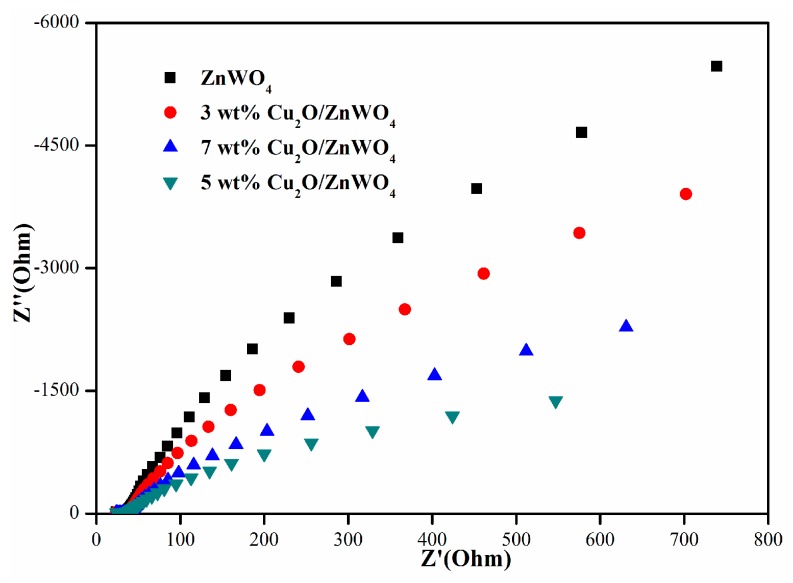
Electrochemical Impedance Spectra (EIS) plots of pure ZnWO_4_ and as-prepared various Cu_2_O/ZnWO_4_ samples irradiated with visible light.

To further test the transport rate of the electrons more intuitively, the electrochemical impedance spectra (EIS) of the prepared materials was carried out. The size of the Nyquist curve reflects the reaction rate and resistance between grain and grain, grain boundaries, and after polarization. The electrode reaction rate decreases due to a larger radius. Small arc radii imply a faster interface electron transfer for better photocatalytic ability [33]. The EIS response of ZnWO_4_ and Cu_2_O/ZnWO_4_ composites in visible light irradiation is shown in Figure 7. Obviously, the semicircular diameter of the composite was smaller than pure ZnWO_4_, which demonstrates that the photo-generated charge can be effectively separated due to the strong interaction between ZnWO_4_ and Cu_2_O. The radius is the smallest when the composite ratio of Cu_2_O is 5 wt %. All these results of EIS, photocurrent, and PL were consistent and illustrated that the introduction of Cu_2_O could greatly accelerate the separation and transfer of photoelectron-hole pairs and improve the degradation performance of composites.

**Figure 8:**
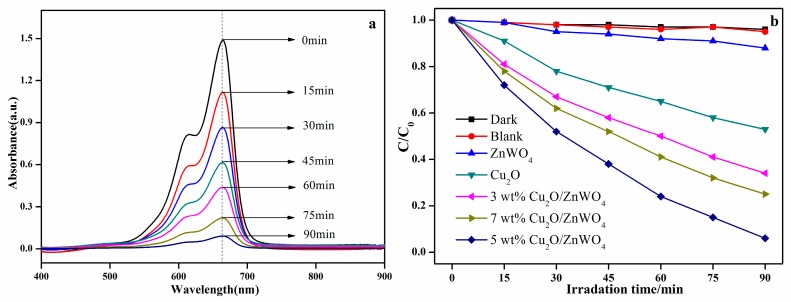
(**a**) UV-Vis spectral changes of methylene blue (MB) aqueous solution in the presence of 5 wt % Cu_2_O/ZnWO_4_ photocatalyst; (**b**) the activity of different catalysts to degrade MB in visible light.

To determine the photocatalytic performance of the catalyst, the activity of degradation MB by ZnWO_4_, Cu_2_O, and Cu_2_O/ZnWO_4_ were compared under the same experimental conditions. As seen in Figure 8a, the characteristic absorption peak of MB was 664 nm, and the intensity decreased gradually in the absence of 5 wt % Cu_2_O/ZnWO_4_ composite under visible light irradiation, and finally disappears at 90 min. Figure 8b displays the change of MB concentration (*C*/*C*_0_) against photodegradation time for the prepared catalyst samples. The experimental results show that MB was not degraded in the absence of a photocatalyst or light irradiation. The MB degradation is a photocatalytic process, in which the photocatalysts generate photo-generated electron-hole pairs under illumination and further act on the target degradation product. Pure ZnWO_4_ has almost no photocatalytic activity because it does not respond to visible light. At the same time, the degradation rate of Cu_2_O to MB was only 47%, which was due to the high recombination rate of excited electron-hole pairs [34]. As a comparison, the 5 wt % Cu_2_O/ZnWO_4_ composites could be degraded at about 90% MB solution, which was derived from the synergic effect of the Cu_2_O/ZnWO_4_ composites, and achieved the rapid separation of photogenerated carriers at the heterojunction. Meanwhile, it was observed that a higher amount of Cu_2_O would decrease the photocatalytic activities. At higher content, the Cu_2_O nanoparticles may agglomerate and reduce the specific surface area of the composites. Additionally, extra Cu_2_O particles may become the new photogenerated recombination centers and, thereby, reduce the effective photogenerated pairs. Then the particles may inhibit the composite photocatalytic performance. All these results were consistent with the PL, photocurrent, and EIS test results of the composites. Therefore, the 5 wt % Cu_2_O/ZnWO_4_ composite exhibited better photocatalytic activity. 

**Figure 9:**
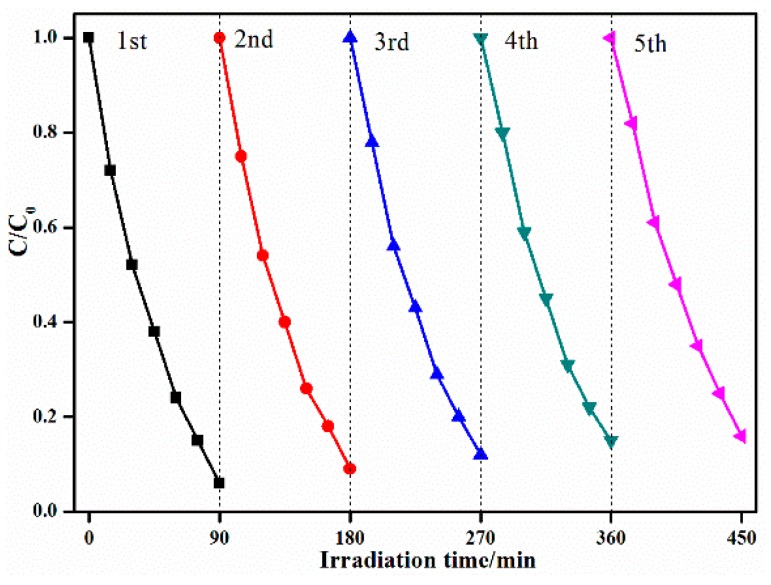
Stability investigation of MB photocatalytic degradation over Cu_2_O/ZnWO_4._

Catalyst stability and reusability are also very important from the viewpoint of the catalyst’s practical applications. The photocatalytic activity of the 5 wt % Cu_2_O/ZnWO_4_ composites in the degradation of MB was studied in consecutive cycles under the same conditions (as seen in Figure 9). The degradation efficiency was slightly decreased after five cycles and the final degrade rate was approximately 83%, which indicates that the Cu_2_O nanoparticles modified in ZnWO_4_ surface preparation of composite photocatalyst could improve the recycling performance of the catalyst and the practical application value. It could act as a potential photocatalyst for water pollution.

**Figure 10:**
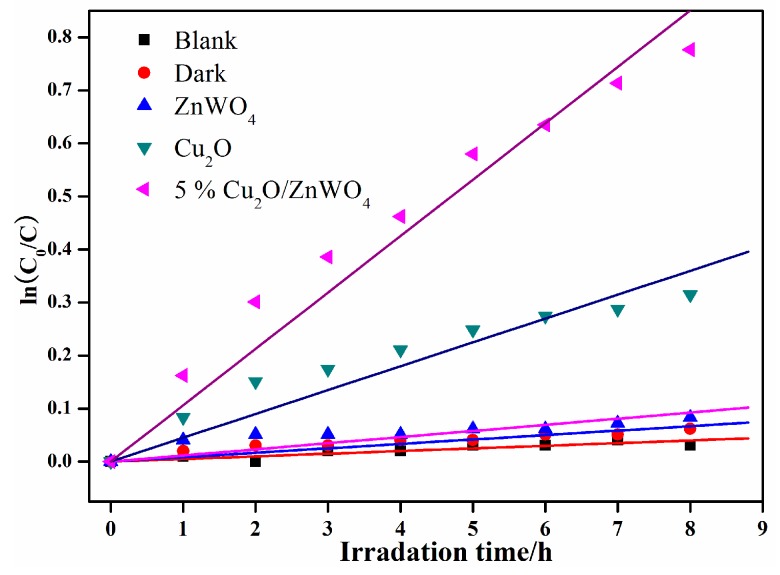
Linear relationship between ln(*C*_0_/*C*) and light time of phenol degradation.

In order to further study the photocatalytic degradation performance of the composites, phenol was used as the degradation object, and the same conditions were tested. It can be seen from Figure 10 that the photolysis of the phenol solution in the blank condition is negligible, and the adsorption capacity of the composite material to the phenol in the dark environment is also very limited. In contrast, the Cu_2_O/ZnWO_4_ photocatalysts exhibit higher catalytic degradation properties for phenol solution. The 5 wt % Cu_2_O/ZnWO_4_ composite photocatalyst exhibits better photocatalytic performance, which was much higher than pure Cu_2_O and ZnWO_4_. This is due to the composite having a bigger advantage than Cu_2_O and ZnWO_4_. More visible light responses can be excited to produce more photogenerated pairs and the photogenerated pairs can be effectively separated between the two composite interfaces, which ultimately promote the photocatalytic performance.

**Figure 11:**
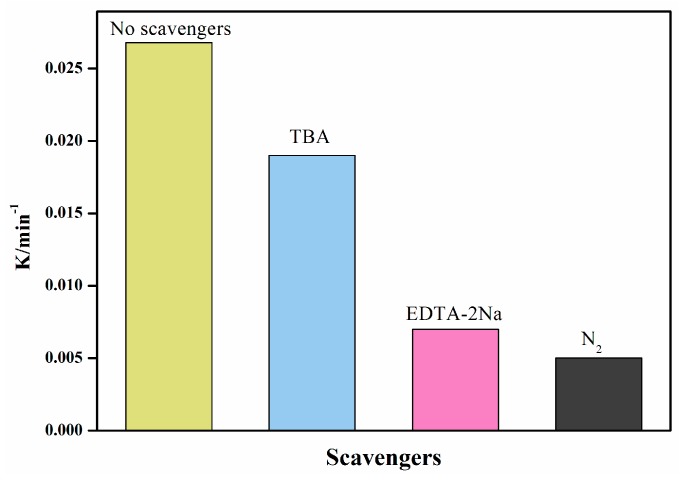
First-order rate constant values of photogenerated active species trapped in the system of photocatalytic degradation of MB by Cu_2_O/ZnWO_4_ under visible light.

Quenching experiments explain the photodegradation process. In the free radical capture experiments of photodegradation, tert-butanol (TBA) and EDTA-2Na were used as capture agents for ·OH and h^+^, respectively [30]. Meanwhile, during the course of the experiment, nitrogen was used to remove oxygen to test whether the superoxide free radicals were active species during the degradation process. As seen in Figure 11, the photodegradation efficiency was significantly suppressed when the EDTA-2Na and in the N_2_-saturated experiment condition was added, which indicates that the h^+^ and O_2_^−^ were likely the dominant active species in this process. In contrast, the dissolved ·OH was not the main active species due to the degradation rate constant showing only a slight decline when added to the TBA solution. Therefore, the photodegradation of MB over Cu_2_O/ZnWO_4_ photocatalyst can be mainly associated with direct holes and O_2_^−^ radicals for the photocatalytic degradation. 

The photocatalytic degradation kinetic curve was investigated by the first-order simplification of Langmuir-Hinshelwood (L-H) kinetics, which is well established for photocatalysis at low initial pollutant concentrations [31]. The relevant equation is as follows:

$$\ln\frac{C_{0}}{C}=kt$$

where *C*_0_ and *C* are the concentrations of dye in solution at times 0 and *t*, respectively, and kapp is the apparent first-order rate constant (min^−1^). The *k* value is obtained from the gradient of the graph of ln(*C*/*C*_0_) versus time (*t*). Based on the characterization analysis and the experimental data, the mechanism of the high photocatalytic activities of Cu_2_O/ZnWO_4_ composites is discussed. Since the light absorption extends from ultraviolet light to visible light, there is no doubt that more visible light absorption can stimulate the generation of more photo-generated carriers, which could be effectively separated between the two composite interfaces because of the match level structure and ultimately promote the photocatalytic performance. According to the conduction band (CB) and valence band (VB) potentials of Cu_2_O (−1.2 and 0.7 eV, respectively [22]) and those of ZnWO_4_ (−0.8 and 2.9 eV, respectively [10]), it can be seen that Cu_2_O/ZnWO_4_ composites have matching energy levels. The staggered energy level structure contributes to the fast separation of photo-generated carriers.

**Figure 12:**
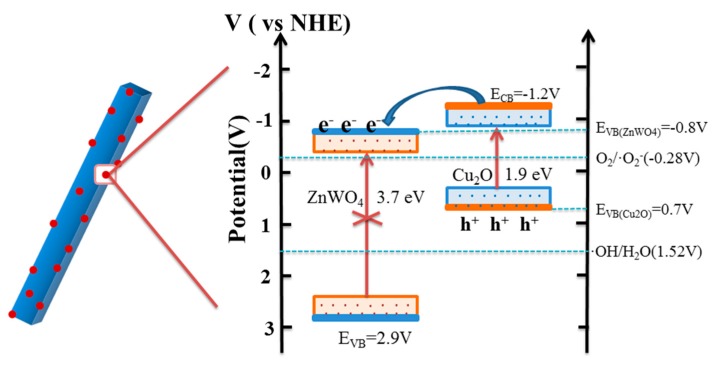
Schematic illustration of photo-generated carriers’ transportation for Cu_2_O/ZnWO_4_ composite.

The possible mechanism of improved photocatalytic performance of Cu_2_O/ZnWO_4_ composite formed by Cu_2_O nanoparticles loading on ZnWO_4_ was deduced based on above experimental results, as seen in Figure 12. This can be explained as follows: Under visible irradiation, Cu_2_O could stimulate the generation of photogenerated hole pairs while the electrons on the conduction band can quickly migrate to the conduction band of ZnWO_4_ at the close interface of the composites since the conduction band of Cu_2_O is more negative than the conduction band of ZnWO_4_. Then the electrons transferred to the ZnWO_4_ react with the adsorbed oxygen molecules to produce O_2_^−^ radicals and participate in the photocatalytic oxidation degradation and the organic pollution process photocatalytic reaction. Simultaneously, after the transition stay in the Cu_2_O valence band hole, another active species can directly oxidize organic pollutants involved in the reaction. Furthermore, this photo-assisted electron transfer method can effectively avoid the photo-generated carrier recombination, improve the utilization of the quantum pair, and promote the photocatalytic activity of the composite. This is consistent with the PL, photoelectric experiment results, and the active species of the quenching experiment.

## 4. Conclusions

The surface of ZnWO_4_ nanorods was decorated with Cu_2_O nanoparticles (Cu_2_O/ZnWO_4_) prepared through the precipitation method. The 5 wt % Cu_2_O/ZnWO_4_ composites displayed the best photocatalytic performance under visible light irradiation and the degradation rate of MB solution was 91%. The improvement in the photocatalytic performance of Cu_2_O/ZnWO_4_ composites due to the Cu_2_O nanoparticles not only promoted the absorption and utilization of visible light, but also facilitated the migration of photogenerated charge carriers due to the matched level structure and the intimately contacted interface. In addition, the O_2_^−^ and holes were predicted to be the main active species in the photocatalytic degradation process based on free-radical scavenging experiments. In conclusion, the Cu_2_O/ZnWO_4_ composite is a highly efficient and stable photocatalyst that can effectively degrade organic pollutants in order to protect the environment.
